# Efficacy and Safety of Folisurge, a Biosimilar Recombinant Follicle-Stimulating Hormone, Versus Gonal-F in Assisted Reproductive Technology Practice in India: A Real-World Study

**DOI:** 10.7759/cureus.98622

**Published:** 2025-12-07

**Authors:** Shashank Sanagoudar, Vipin Chandra, Shipra Nigam, Anjali Gahalan, Ritu Punhani, Nihita Pandey, Sara Zaidi, Jyoti Panday, Nihar Ranjan Bhoi, Walmik Mistari, Isha Suwalka, Nitasha Gupta

**Affiliations:** 1 Department of Reproductive Medicine, Indira IVF Hospital Private Limited, Udaipur, IND; 2 Department of Clinical Research and Operations, Indira IVF Hospital Private Limited, Udaipur, IND; 3 Department of Reproductive Medicine, Indira IVF Hospital Private Limited, Shahdara, IND; 4 Department of Reproductive Medicine, Indira IVF Hospital Private Limited, Allahabad, IND; 5 Department of Reproductive Medicine, Indira IVF Hospital Private Limited, New Delhi, IND; 6 Department of Reproductive Medicine, Indira IVF Hospital Private Limited, Kolkata, IND; 7 Department of Reproductive Medicine, Indira IVF Hospital Private Limited, Dadar, IND; 8 Department of Reproductive Medicine, Indira IVF Hospital Private Limited, Ghaziabad, IND; 9 Department of Clinical Lab and Operations, Indira IVF Hospital Private Limited, Udaipur, IND; 10 Department of Research and Publication, Indira IVF Hospital Private Limited, Udaipur, IND

**Keywords:** antral follicle count (afc), assisted reproductive technology (art), body mass index (bmi), controlled ovarian stimulation (cos), recombinant human follicle-stimulating hormone (r-hfsh), serum anti-mullerian hormone

## Abstract

Objective: This study was conducted to compare the efficacy and safety of Folisurge, a biosimilar recombinant follicle-stimulating hormone (r-FSH), with Gonal-f, an innovator r-FSH, in patients undergoing assisted reproductive technology (ART).

Methods: In this non-interventional, investigator-initiated, real-world, retrospective study, females aged 21-40 years with a body mass index (BMI) ranging between 17 and 34.9 kg/m^2^, with serum anti-Müllerian hormone (AMH) more than 1.2 ng/mL, undergoing controlled ovarian stimulation (COS), and those having received Folisurge or Gonal-f were included. The primary outcome was the mean number of metaphase II (MII) oocytes retrieved in each group. Secondary outcomes included the mean number of embryos formed and serum β-human chorionic gonadotropin (β-hCG) positive rate in each group.

Results: A total of 3090 women were included, out of which 1210 and 1880 received Folisurge and Gonal-f, respectively. The baseline demographics were comparable in the two groups. The mean antral follicle count (AFC) was significantly higher in the Folisurge vs. the Gonal-f group (23.8 vs. 20.6, p < 0.001). Also, the mean (SD) number of oocytes retrieved was significantly higher in the Folisurge vs. the Gonal-f group (18.1 (9.4) vs. 15.5 (8.3), p < 0.001). The mean (SD) number of MII oocytes retrieved with Folisurge was significantly higher than Gonal-f (13 (7.5) vs. 10.9 (6.6), p < 0.001). Also, the mean (SD) number of embryos formed was also higher in the Folisurge vs. the Gonal-f group (4.9 (4.1) vs. 4.7 (4.1), p = 0.022). The serum β-hCG positive rate was similar between the two groups (75.5% vs. 76.2%, p = 0.667). No major safety concerns were observed in the study.

Conclusion: Folisurge was comparable to Gonal-f in terms of serum β-hCG positive rate. The higher yield of oocytes and embryos in the Folisurge group should be interpreted in the context of its higher baseline AFC, which may have influenced these results. No major safety concerns were observed.

## Introduction

As per the World Health Organization (WHO), in the developing nations, one in four couples suffers from infertility [1]. A study suggested that in India, about 15-20 million couples per year suffer from infertility, and approximately 25% of the world’s infertile couples belong to India [2,3]. Infertility takes an immense toll on the social, economic, and mental well-being of many couples in India.

Assisted reproductive technology (ART) has proved to be immensely beneficial to such couples with improved success rates in the current scenario. Follicle-stimulating hormone (FSH) in various formulations has been the backbone of ART cycles for controlled ovarian stimulation (COS) since the time of inception [4]. Initially, the source of FSH was the urine of menopausal women. However, the drawbacks of urine sources are batch-to-batch variability, risk of transmission of infection, and lack of consistency in results [5]. The advent of recombinant follicle-stimulating hormone (r-FSH) has revolutionized the outcome of stimulation as the oocytes respond more consistently to r-FSH and there are fewer inter-batch variations in their activity [5,6].

Gonal-f was the first recombinant follitropin-alpha (r-FSH α) manufactured by Merck Serono (Darmstadt, Germany). It was developed in 1988 but was licensed in the European Union in 1995 and received the United States Food and Drug Administration (USFDA) approval in 2004 [7]. Gonadotrophin therapy is a significant proportion of ART treatment costs, which in emerging economies like India [8] are out-of-pocket expenses for the patients [8]. Thus, the introduction of r-FSH biosimilars has the potential to reduce the treatment costs globally. This opened the doors for biosimilars, which are compounds similar in function, although not identical to the originator. Many countries have approved different biosimilars for use in ART [8-10].

Folisurge (manufactured by Intas Pharmaceuticals Ltd, Ahmedabad, India), a recombinant human follicle-stimulating hormone alpha (h-FSH-α) biosimilar, was approved by the Drugs Controller General of India (DCGI) in May 2013. However, any study with a direct comparison of Folisurge and Gonal-f in a real-world setting is not available. Hence, this real-world, non-interventional, retrospective, investigator-initiated study was conducted to compare the efficacy and safety of Folisurge versus Gonal-f in patients undergoing ART.

## Materials and methods

Study design

This was a non-interventional, investigator-initiated, retrospective, real-world, observational study, conducted at Indira IVF, a private chain of ART hospitals, at multiple locations across India, from April 2019 to June 2021. The study utilized patient data extracted from the centralized hospital information system, which maintains comprehensive electronic medical records across the institution. Patient records from April 2019 to June 2021 were considered from the hospital's central data repository.

All females undergoing COS in in-vitro fertilization (IVF), followed by frozen embryo transfer (FET) cycles between the age groups of 21 and 40 years, with a body mass index (BMI) ranging between 17 and 34.9 kg/m2, and an anti-Müllerian hormone (AMH) level more than 1.2 ng/mL, were included in the study.

The research did not include data from participants undergoing the following treatments: (1) cycles with sperm obtained by microscopic epididymal sperm aspiration (MESA), testicular sperm extraction (TESE), testicular sperm aspiration (TESA), and percutaneous epididymal sperm aspiration (PESA); (2) cycles with donor oocytes; (3) cycles with genetic preimplantation diagnosis; (4) cycles with female fertility preservation; (5) ovarian stimulation with follitropin alfa in conjunction with another gonadotropin [8]. Four patient groups were examined for Folisurge and Gonal-f based on their initial r-FSH dosages: less than 150 IU, 150-224 IU, 225-299 IU, and more than 300 IU.

The study protocol and related documents were reviewed and approved by the Indira IVF Hospital Institutional Ethics Committee (Reg No: ECR/1773/Inst/PB/2023), The study was conducted in accordance with the ethical principles that have their origin in the Declaration of Helsinki and in accordance with the International Conference on Harmonization’s Good Clinical Practice (ICH-GCP) guidelines [11], New Drugs and Clinical Trial (NDCT) Rules - 2019 [12], Indian Council of Medical Research (ICMR) Guidelines for Biomedical Research (2017) [13], applicable regulatory requirements, and in compliance with the protocol. This was a retrospective study without patient identifiers; hence, the informed consent of patients was not taken.

Study assessments

The primary outcome was the mean number of metaphase II (MII) oocytes retrieved. Secondary outcomes included the mean number of embryos formed and serum β-human chorionic gonadotropin (β-hCG) positive rate in each group. Only variables consistently gathered by the various centers were used for secondary endpoints and demographic data. These variables were the number of days of r-FSH stimulation, the total dosage of r-FSH delivered, the number of oocytes retrieved, the number of MII oocytes, and the number of embryos produced.

Statistical analysis

Because the goal of this study was descriptive, no formal sample size calculations were performed; instead, the sample size was determined by assuring sufficient numbers to explain the efficacy of Folisurge in routine usage. Descriptive data are supplied with continuous data expressed as mean ± SD or, if non-normally distributed, as median [8]. All statistical analyses were carried out using SAS® version 9.4 (SAS Institute, Cary, NC). All statistical tests had to be two-sided at α = 0.05, unless otherwise noted.

## Results

**Figure 1:**
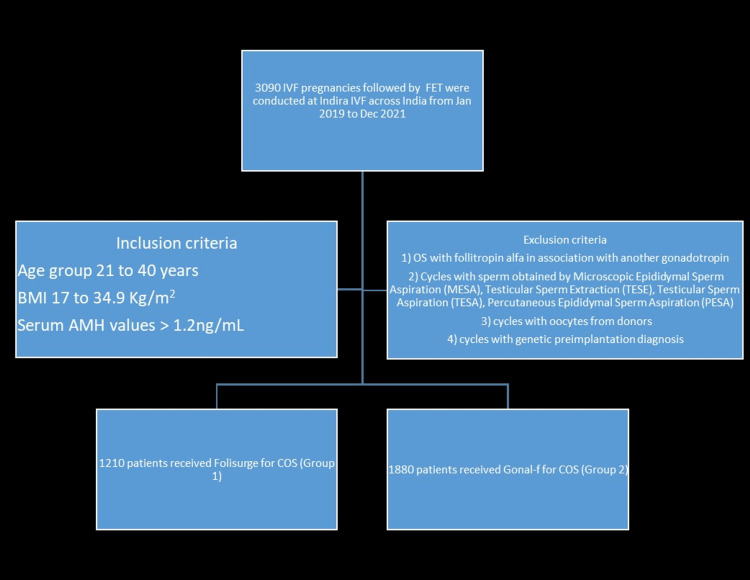
Patient enrollment flowchart. IVF: in-vitro fertilization; FET: frozen embryo transfer; AMH: anti-Müllerian hormone; OS: ovarian stimulation; COS: controlled ovarian stimulation.

A total of 3090 women were studied, out of which 1210 received Folisurge, and 1880 received Gonal-f as described in the Consolidated Standards of Reporting Trials (CONSORT) diagram (Figure 1). The baseline characteristics were comparable between the two groups (Table 1). In the Folisurge group, the mean antral follicle count (AFC) was significantly higher than in the Gonal-f group (23.8 vs. 20.6, p < 0.001). The mean sperm count was significantly lower in the Folisurge group than in the Gonal-f group (43.1 million/mL vs. 47.2 million/mL, p < 0.001). Also, the mean sperm motility was significantly lower in the Folisurge group than in the Gonal-f group (39.0% vs. 40.7%, p < 0.001).

**Table 1:** Basic demographic profile of all participants included in the study for controlled ovarian stimulation via Folisurge or Gonal F. AMH: anti-Müllerian hormone; AFC: antral follicle count.

	Overall (n = 3090)	Folisurge group (n = 1210)	Gonal-f group (n = 1880)	
Characteristics				p-value
Age in years, mean (SD)	29.6 (3.9)	29.1 (3.6)	29.9 (4.0)	0.452
BMI in kg/m^2^, mean (SD)	24.7 (3.9)	24.8 (3.9)	24.6 (3.9)	0.501
Markers of ovarian reserve				
Serum AMH in ng/mL, mean (SD)	4.5 (2.4)	4.8 (2.4)	4.3 (2.4)	0.062
AFC, mean (SD)	21.8 (9.8)	23.8 (10.1)	20.6 (9.3)	<0.001
Husband/donor's age				
Husband/donor's age in years, mean (SD)	33.6 (4.24)	33.4 (4.1)	33.7 (4.3)	0.064
Semen parameters				
Sperm count in million/mL, mean (SD)	45.6 (25.9)	43.1 (26.9)	47.2 (25.1)	<0.001
Sperm motility (%), mean (SD)	40.0 (20.8)	39.0 (21.7)	40.7 (20.1)	<0.001
Normal sperm morphology (%), mean (SD)	2.42 (1.4)	2.13 (1.5)	2.6 (1.4)	0.106

In both groups, patients were administered r-FSH, and the starting dose ranged from 150 IU to 375 IU. Based on the starting dosage of r-FSH, there were no differences seen between the two groups in terms of age, BMI, and serum AMH levels (Table 2). The AFC in the Folisurge group was higher than the Gonal-f group in most of the dosage strengths used. The sperm count, motility, and morphology were lower in the Folisurge group vs. the Gonal-f group in most of the dosages studied.

**Table 2:** Baseline demographic profile of participants based upon the starting dose of gonadotropin used for controlled ovarian stimulation (subgroup-wise). AMH: anti-Müllerian hormone; AFC: antral follicle count.

	Folisurge (N = 1210)				Gonal-f (N = 1880)			
Starting dose	≤150 IU (n = 231)	151–224 IU (n = 202)	225-299 IU (n = 737)	≥300 IU (n = 40)	≤150 IU (n = 704)	151–224 IU (n = 188)	225-299 IU (n = 938)	≥300 IU (n = 50)
Wife's age, mean (SD)	30.8 (4.0)	27.6 (3.3)	29.0 (3.4)	30.6 (3.6)	31.2 (4.2)	28.0 (3.4)	29.3 (3.7)	29.5 (3.4)
BMI, mean (SD)	24.0 (3.9)	23.8 (3.2)	25.1(3.9)	26.7 (4.3)	24.4 (3.8)	23.4 (3.6)	24.9 (3.9)	26.7 (4.4)
AMH, mean (SD)	3.3 (2.3)	7.1 (1.5)	4.7 (2.2)	4.3 (1.0)	3.0 (2.0)	7.1 (1.5)	4.7 (2.1)	4.2 (1.0)
AFC, mean (SD)	18.0 (9.7)	29.8 (9.4)	24.1 (9.4)	21.0 (10.0)	15.0 (7.3)	28.9 (9.1)	23.1 (8.4)	21.8 (7.2)
Husband/donor's age in years, mean (SD)	34.3 (4.0)	32.0 (3.9)	33.4 (4.1)	33.9 (3.6)	34.6 (4.5)	32.3 (3.9)	33.3 (4.1)	35.2 (4.6)
Sperm count (M/mL), mean (SD)	38.6 (25.5)	42.5 (25.3)	44.6 (27.8)	44.6 (24.5)	46.9 (26.3)	47.4 (24.5)	47.4 (24.3)	43.6 (26.9)
Sperm motility (%), mean (SD)	35.2 (22.2)	40.9 (22.0)	39.6 (21.6)	40.3 (18.6)	40.2 (19.8)	40.9 (20.2)	41.1 (20.3)	39.1 (20.8)
Sperm morphology (%), mean (SD)	1.5 (1.4)	1.8 (1.5)	2.4 (1.5)	2.7 (1.3)	2.4 (1.4)	2.7 (1.3)	2.7 (1.4)	2.8 (1.3)

The mean number of days of stimulation was similar in both groups across different starting doses (Table 3). Also, there was no major change in the starting dose and daily dose across different subgroups for both the Folisurge and Gonal-f groups. The mean (SD) number of oocytes retrieved was significantly higher in the Folisurge group vs. the Gonal-f group (8.1 (9.4) vs. 5.5 (8.3), p < 0.001) (Table 4).

**Table 3:** Details of ovarian stimulation cycles (days of stimulation, daily dose, and total dose of r-FSH used) according to the starting dose of Folisurge or Gonal-f. COS: controlled ovarian stimulation; r-FSH: recombinant follicle-stimulating hormone.

Characteristics (n = 3090)	Starting dose of Folisurge (n = 1210)				Starting dose of Gonal-f (n = 1880)			
	<=150 IU	151-224 IU	225-299 IU	>=300 IU	<=150 IU	151-224 IU	225-299 IU	>=300 IU
	Days of stimulation, mean (SD)	11.5 (1.4)	11.0 (1.4)	11.1 (1.3)	11.6 (1.6)	11.5 (1.3)	11.3 (1.4)	11.1 (1.3)	11.3 (1.5)
Daily dose of r-FSH used for COS, mean (SD)	150.0 (0.0)	187.5 (0.0)	229.6 (14.4)	309.4 (25.1)	150.0 (0.0)	187.5 (0.0)	228.0 (11.9)	307.5 (22.7)
Total dose of r-FSH used, mean (SD)	1718.8 (216.6)	2065.3 (266.2)	2538.2 (338.8)	3579.4 (565.6)	1731.2 (201.5)	2116.0 (269.3)	2533.9 (329.4)	3454.5 (452.4)

**Table 4:** Outcome of oocyte retrieval (in terms of number and quality of oocytes retrieved) and embryos (in terms of number and quality of embryos formed) in Folisurge and Gonal-f groups. MII: metaphase II; OSI: ovarian sensitivity index; FOI: follicle-to-oocyte index; FORT: follicular output rate.

Characteristics	All patients (n = 3090)	Folisurge patients (n = 1210)	Gonal-f patients (n = 1880)	P-value
Number of oocytes expected, mean (SD)	15.6 (8.4)	17.2 (9.2)	14.5 (7.6)	<0.001
Number of oocytes retrieved, mean (SD)	16.5 (8.8)	18.1 (9.4)	15.5 (8.3)	<0.001
Oocyte retrieval rate, mean (SD)	114.1 (50.0)	112.6 (48.3)	115 (51.1)	0.204
Number of metaphase II oocytes, mean (SD)	11.8 (7.1)	13 (7.5)	10.9 (6.6)	<0.001
MII rate, mean (SD)	70.5 (14.8)	71.4 (14.5)	69.9 (14.9)	0.001
Embryo details				
Number of embryos formed, mean (SD)	4.8 (4.1)	4.9 (4.1)	4.7 (4.1)	0.022
Blastocyst rate, mean (SD)	43.9 (28.5)	42.3 (27.6)	44.9 (29.0)	0.008
Good blastocyst rate, mean (SD)	30.0 (21.8)	28.2 (20.9)	31.2 (22.3)	<0.001
Number of embryos transferred, mean (SD)	1.9 (0.4)	1.9 (0.4)	1.9 (0.4)	0.078
Key performance indicators of ovarian stimulation				
OSI, mean (SD)	184.7 (140.9)	176.5 (147.3)	189.9 (136.4)	<0.001
FOI, mean (SD)	74.3 (16.3)	74.6 (16.3)	74.2 (16.2)	0.408
FORT, mean (SD)	71.3 (19.1)	72.4 (19.4)	70.7 (18.8)	0.004

The mean (SD) number of MII oocytes retrieved with Folisurge was significantly higher than Gonal-f (13 (7.5) vs. 10.9 (6.6), p < 0.001). The mean (SD) number of embryos formed was also higher in the Folisurge vs. Gonal-f group (4.9 (4.1) vs. 4.7 (4.1), p = 0.022) (Table 4). On comparing the key performance indicators of ovarian stimulation, the mean (SD) follicular output rate (FORT) was significantly higher in the Folisurge vs. the Gonal-f group (72.4 (19.4) vs. 70.7 (18.8), p = 0.004). Further analysis of the oocyte and embryo outcomes according to the starting dose suggested that more number of oocytes were retrieved in the Folisurge vs. the Gonal-f groups across most of the dosages studied (Table 5). On comparison of the number of MII oocytes formed, Folisurge performed better in all doses except ≥ 300 IU. It was found that Folisurge formed more embryos than Gonal-f across most of the doses studied.

**Table 5:** Outcome of oocyte retrieval (in terms of number and quality of oocytes retrieved) and embryos (in terms of number and quality of embryos formed) in the Folisurge and Gonal-f groups according to the starting dose. MII: metaphase II; OSI: ovarian sensitivity index; FOI: follicle-to-oocyte index; FORT: follicular output rate.

Characteristics (n = 3090)	Folisurge (n = 1210)				Gonal-f (n = 1880)			
	<=150 IU	151-224 IU	225-299 IU	>=300 IU	<=150 IU	151-224 IU	225-299 IU	>=300 IU
	Mean (SD)							
Outcomes of oocyte retrieval according to the starting dose of Folisurge or Gonal-f								
Oocytes expected	13.3 (9.5)	21.8 (9.7)	17.3 (8.5)	14.8 (7.8)	10.1 (5.2)	20.7 (8.6)	16.5 (7.3)	14.1 (6.1)
Oocytes retrieved	13.3 (9.1)	23.4 (9.4)	18.2 (8.8)	15.4 (9.0)	11.0 (6.5)	22.8 (8.6)	17.3 (7.8)	16.5 (7.1)
Oocyte retrieval rate	110.2 (51.9)	115.4 (45.4)	112.5 (48.1)	113.9 (45.1)	116.2 (52.6)	118.6 (50.1)	112.4 (48.4)	133.0 (75.2)
Number of MII oocytes	9.9 (6.8)	16.8 (7.5)	13.1 (7.3)	11.1 (7.6)	7.8 (5.1)	16.7 (7.5)	12.1 (6.3)	11.3 (5.1)
MII oocyte rate	75.2 (15.6)	72.2 (14.2)	70.2 (13.8)	68.5 (16.6)	70.2 (15.4)	72.4 (13.3)	69.3 (14.9)	69.3 (13.1)
Embryo details								
Embryos formed	4.0 (3.7)	5.7 (4.4)	4.9 (3.9)	6.1 (6.1)	3.7 (3.1)	7.7 (5.4)	4.8 (4.2)	4.0 (2.9)
Blastocyst rate	47.5 (28.7)	38.0 (27.3)	41.3 (27.0)	53.3 (27.5)	51.0 (31.3)	47.1 (27.5)	40.2 (26.7)	38.6 (28.0)
Good blastocyst rate	34.8 (24.7)	23.2 (16.9)	27.1 (20.0)	35.4 (22.7)	35.7 (25.4)	28.6 (18.8)	28.5 (20.0)	28.8 (19.5)
Number of embryos transferred	1.8 (0.4)	1.9 (0.3)	1.9 (0.4)	1.9 (0.5)	1.8 (0.4)	1.9 (0.4)	1.9 (0.4)	1.9 (0.5)
Key performance indicators of ovarian stimulation								
OSI	215.8 (226.8)	102.5 (42.4)	177.7 (120.3)	301.8 (182.0)	218.7 (164.2)	107.8 (50.5)	180.8 (108.2)	263.7 (218.9)
FOI	71.4 (21.5)	77.9 (15.0)	74.8 (14.6)	73.4 (15.5)	72.6 (18.2)	78.7 (15.2)	74.4 (14.7)	74.6 (15.1)
FORT	71.0 (21.0)	73.3 (19.4)	72.6 (18.8)	71.5 (20.6)	68.4 (19.1)	72.3 (19.3)	72.3 (18.0)	66.3 (23.0)

**Figure 2:**
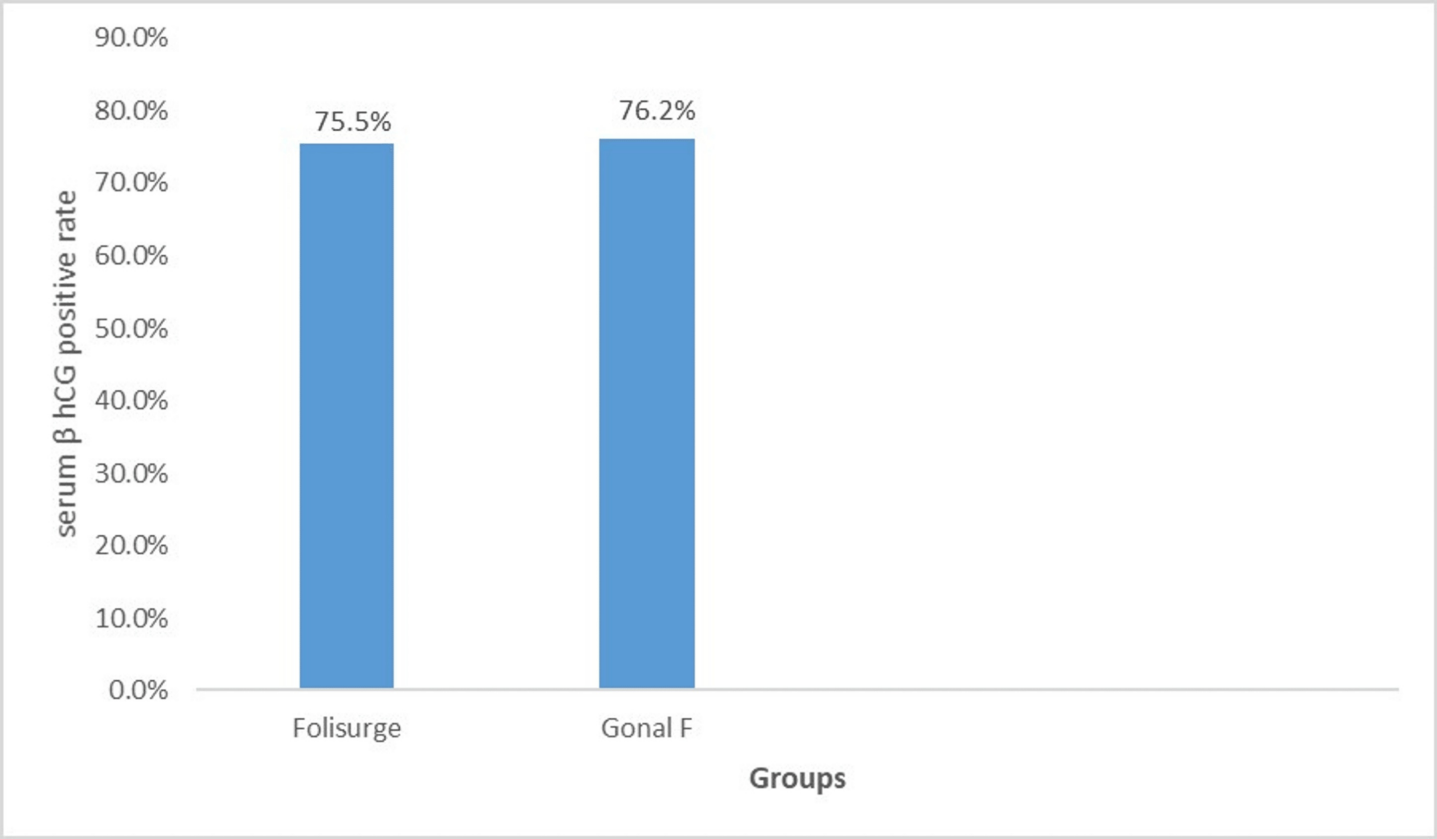
Serum β-hCG positive rate in each of the groups of participants receiving either Folisurge or Gonal-f. β-hCG: β-human chorionic gonadotropin.

**Figure 3:**
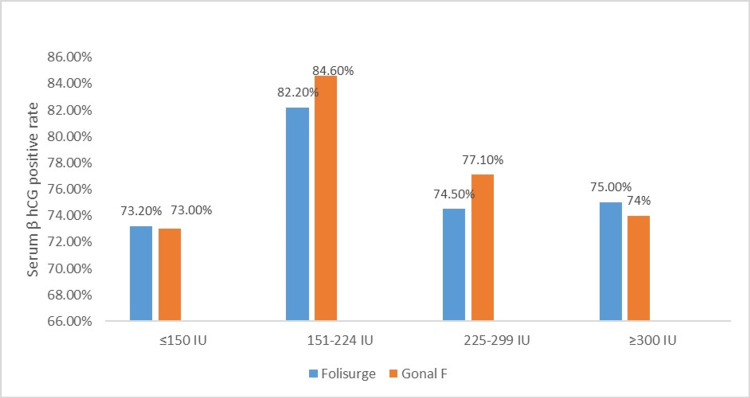
Serum β-hCG positive rate according to starting dose in each of the groups of participants receiving either Folisurge or Gonal-f. β-hCG: β-human chorionic gonadotropin.

The serum β-hCG positive rate was similar in both groups (75.5% vs. 76.2%; p = 0.667) (Figure 2). On further subgroup analysis according to the starting dose, there was not much variation among the two groups in the various doses studied (Figure 3). A review of patient records revealed no documented cases of severe ovarian hyperstimulation syndrome (OHSS) or other major adverse events leading to hospitalization in either group.

## Discussion

In this current study, we examined the variance of the r-FSH beginning dose, a significant and contentious issue, and reported on the first usage of a biosimilar r-FSH, Folisurge, against the innovator Gonal-f in 106 ART sites throughout India. In terms of serum hCG-positive rates in actual clinical practice, this study also demonstrates that Folisurge and the pioneer Gonal-f are equally effective. Homogeneity of the comparison populations and intriguing information on the connection between FSH dose, conditions of usage of these doses, and outcomes are provided by the analyses based on the initial r-FSH dose [8].

Manzi et al. (2022) compared the structural features of Gonal-f vs. r-FSH biosimilar preparations in non-European regions, and Folisurge was the only biosimilar chosen from India. The biosimilars were compared with respect to glycosylation, macro- and micro-heterogeneity, sialylation, and immunogenicity. It was found that Folisurge was aligned with the Gonal-f across all N-glycosylation sites, and it had a higher degree of O-acetylation vs. Gonal-f, which is a representative of a lower risk of immunogenicity [14]. Despite minor changes in the structure of biosimilar preparations vis-à-vis the reference product, ultimately, the clinical efficacy and safety are of paramount importance. This study was conducted to obtain information on the comparison of Folisurge vs. Gonal-f on the efficacy aspect.

The number of oocytes retrieved is considered a surrogate marker of the efficacy of the medication and is often used in different studies [15]. Also, considering the fact that the number of MII oocytes retrieved may have a bearing on the clinical pregnancy outcomes [16], the number of MII oocytes retrieved was considered as the primary outcome in the current study. The mean (SD) number of MII oocytes retrieved in the Folisurge group was significantly higher than the Gonal-f group (13.0 (7.5) vs. 10.9 (6.6); p < 0.001). Also, the MII oocyte rate was significantly higher in the Folisurge group versus the Gonal-f group (71.4% vs. 69.9%; p = 0.001), suggesting that Folisurge was better in terms of the quality of oocytes retrieved per patient. In a similar study conducted by Barakhoeva et al. (2019) comparing Primapur (recombinant FSH alfa biosimilar) with Gonal-f showed that the mean number of MII oocytes retrieved was similar (Primapur = 9.6 (6.3) vs. Gonal-f = 9.9 (5.6); p = 0.617). This shows that biosimilars can also produce equal efficacy vis-à-vis originator [17].

It was observed that the mean (SD) number of embryos formed in the Folisurge group was significantly higher than the Gonal-f group (4.9 (4.1) vs. 4.7 (4.1); p = 0.022). Further, while comparing the subgroups based on the starting dose, it was found that more embryos were formed with Folisurge than Gonal-f in all dosage groups, except 151-224 IU. Folisurge was comparable to Gonal-f in terms of serum β-hCG positive rate (75.5% vs. 76.2%; p = 0.667). On further subgroup analysis, according to the starting dose, there was not much variation between the two drugs. Similar results were observed by Barakhoeva et al. (2019) in terms of serum β-hCG positive rate among Primapur and Gonal-f (34.7% vs. 36.7%; p = 0.833) [17].

The age, BMI, weight, polycystic ovarian syndrome, smoking history, severe endometriosis, prior ovarian response, prior pelvic surgery, AFC, ovarian volume, ovarian stromal blood flow, serum AMH, serum FSH, serum luteinizing hormone, serum estradiol, serum inhibin B, serum testosterone, and various dynamic tests of ovarian reserve are all important factors to consider when determining the starting dose of r-FSH. [8] The goal is to achieve an adequate response to ovarian stimulation while minimizing the risk of OHSS. The r-FSH starting dosage may be determined with the use of a variety of prediction algorithms [8,18]. The prescribing information of Gonal-f mentions that there have been a few cases of severe adverse effects, including hypersensitivity. This highlights the need for comprehensive safety monitoring with detailed surveillance for rare and atypical reactions (both clinical and biochemical) to ensure a complete understanding of its safety profile [19]. Furthermore, because prognostic variables combine in a complicated way, there is currently no universally accepted method for determining the first r-FSH alpha dosage for every patient; instead, the treating physician's professional opinion is eventually used [8]. In our study, when the subgroup analysis based on the starting dose of FSH was conducted between the Folisurge and Gonal-f groups, the results were comparable in both groups.

Limitations

Many bodies, such as the European Society of Human Reproduction and Embryology (ESHRE) and the International Committee Monitoring Assisted Reproductive Technologies (ICMART), consider live birth and cumulative live birth as the most relevant outcome for infertility treatment [18,20]; however, the same could not be captured in the current study. The lack of live birth rate data is a significant limitation, as it prevents full assessment of the comparative clinical efficacy of the two treatments. Being a real-world study, structured adverse event reporting was missing. Another limitation of our study was its retrospective nature. Multicenter, randomized, double-blind, controlled clinical studies are required to compare the clinical outcomes, especially live birth rate, of Folisurge vs. Gonal-f. A pharmacoeconomic analysis will be helpful in further evaluating the cost-effectiveness of biosimilars.

## Conclusions

Folisurge was comparable to Gonal-f in terms of serum β-hCG positive rate. The higher yield of oocytes and embryos in the Folisurge group should be interpreted in the context of its higher baseline AFC, which may have influenced these results. No major safety concerns were observed.
